# Non-Deceptive Placebos Can Promote Acts of Kindness: A Randomized Controlled Trial

**DOI:** 10.3390/bs13090703

**Published:** 2023-08-23

**Authors:** Anne Schienle, Isabella Unger

**Affiliations:** Department of Clinical Psychology, University of Graz, 8010 Graz, Austria

**Keywords:** non-deceptive placebo, open-label-placebo, acts of kindness, pleasantness, satisfaction with life

## Abstract

Placebos have often been used to reduce emotional distress but rarely to increase positive feelings. The present study investigated whether a placebo can promote acts of kindness (AoKs) that are associated with emotional well-being. A total of 160 university students were asked to perform an AoK daily for one week. They evaluated their emotional state (feelings of pleasantness, arousal, satisfaction) directly before and after the AoKs. This was monitored via a smartphone app. One group performed each AoK after taking a non-deceptive placebo; the other group received no placebo. Before and after the one-week program, the participants completed three questionnaires that assessed satisfaction with life, positive/negative affect, and flourishing. The participants reported higher pleasantness directly after engaging in an AoK and more satisfaction with life after the program. The motivation to carry out AoKs decreased strongly over the week. However, placebo receivers completed more AoKs than the no-placebo group. The results indicate that placebo treatment can promote the performance of acts of kindness.

## 1. Introduction

Placebos can help to cope with emotional distress, even in cases where people have been told they are receiving a placebo (an inert treatment or substance). Several studies with non-deceptive placebos (also known as open-label placebos: OLPs) have shown that this type of treatment is associated with the reduction in stress-related symptoms (e.g., trouble sleeping) and emotional distress [1,2,3]. In two large studies with electroencephalography, the administration of a non-deceptive placebo (a saline nasal spray) was associated with a reduction in both self-reports and neural measures of emotional distress during the viewing of unpleasant pictures [4,5].

So far, only rarely have placebos been used to promote positive emotions and positive activities. For example, a deceptive placebo (sunflower oil labeled as herbal medicine that helps to focus on one’s inner strengths) increased the frequency of relaxation practice in healthy students during a two-week training [6]. In a study by Gaab et al. [7], one group of healthy participants viewed an animated video showing green dots, along with the placebo suggestion that ‘the color green activates early conditioned emotional schemata’. After three days of treatment, it was found that, when administered in the context of a friendly experimenter-participant relationship, the placebo enhanced mental well-being. Moreover, positive effects of deceptive placebos have been reported in studies on health-promoting activities, such as sports (for a review, see Beedie et al. [8]). Placebos prescribed as ergogenic aids have improved speed and endurance in athletes. Thus, placebos can help to energize and direct behaviors toward a goal. They can increase motivation (see Hyland [9]).

A study on the effects of relaxation training demonstrated that this motivational effect can also be achieved via a non-deceptive placebo [10]. In this experiment, the participants (university students) were randomly assigned to a two-week PMR (progressive muscle relaxation) course with or without daily OLP treatment. The OLP group received sunflower oil provided in a glass bottle with a dropper for oral administration. The label of the bottle stated ‘placebo’. The participants were instructed to take the oil (three drops) directly before each PMR session and that the placebo would support the body’s natural relaxation response. Moreover, information about placebo effects and the mechanisms assumed to be responsible for these effects (i.e., expectation, conditioning) were provided. It was found that the participants of the OLP group completed more PMR exercises than the group without a placebo. However, both groups did not differ in reported exercise-related changes in relaxation levels. Thus, the OLP positively influenced the quantity but not the quality of relaxation training.

The present non-deceptive placebo study aimed to promote engagement in another type of positive behavior that is associated with well-being: acts of kindness (AoKs; [11,12,13,14,15,16]). Kindness includes caring, affection, generosity, consideration, and concern toward others. AoKs are intentional acts undertaken to benefit others [13]. In a study by Chancellor et al. [11], participants performed five acts of kindness for a personalized list of AoK recipients over a period of four weeks. Benefits in well-being for the givers and the recipients were found in both the short-term and the long-term. The recipients reported becoming happier, and the givers reported becoming more satisfied with their lives and less depressed after two months. An earlier investigation by Dunn et al. [17] found that spending money on others improved one’s well-being more than spending it on oneself. Further, Shillington et al. [15] conducted a two-week study with university students and found that the addition of AoKs to a stress-management intervention improved participants’ resilience.

In summary, engaging in AoKs has been shown to be a useful method for increasing subjective well-being (also see meta-analysis by Curry et al. [12]). At the same time, various factors can hinder the performance of AoKs and thus prevent their positive effects (e.g., time pressure). A series of studies by Boothby and Bohns [18] suggested that people refrain from engaging in AoKs because they underestimate the value of their behavior to others. Therefore, to realize the positive effects, it could be helpful to provide potential givers of AoKs with a tool to motivate the carrying out of the act of kindness.

In the present study, participants were randomly assigned to one of two groups that performed a daily AoK for one week, either with or without the OLP treatment. Participants planned the specific acts of kindness, selected the recipients, and evaluated their emotional state before and after the AoKs. It was hypothesized that AoKs would have immediate positive effects (e.g., an increase in reported feelings of pleasantness and satisfaction).

The rating was performed via a smartphone application. Research has shown that apps are helpful tools to monitor compliance and the effects of interventions continuously (e.g., [10]). Thus far, AoK studies have often relied on post hoc ratings (with a time lag between AoK completion and evaluation, e.g., [19]). This may be associated with biased reports since AoKs are socially desired behaviors.

The participants of the present study also answered various questionnaires (Satisfaction with Life Scale, Positive Affect Negative Affect Schedule, Flourishing Scale) before and after the one-week AoK program. It was hypothesized that, compared to the group without OLP, the placebo group would perform AoKs more frequently and would report more positive AoK effects (e.g., an increase in reported positive affect and satisfaction with life).

## 2. Materials and Methods

### 2.1. Participants

A total of 160 University students (125 females, 35 males; mean age: 24.71 years, SD = 3.77) in a master’s psychology program participated in a one-week AoK program. Participants were randomly allocated to the standard program or the program with additional daily OLP treatment (groups: placebo vs. no-placebo). The two groups did not differ in mean age and gender ratio; *p* > 0.50). Exclusion criteria were self-reported diagnoses of mental disorders and intake of psychotropic medication (open response format in an online survey tool).

Before the study, a power analysis was conducted with the software program G*Power 3 [20]. To detect an effect size of 0.25 (0.05 alpha error probability; 0.95 power), a total sample size of *n* = 158 is required for a mixed analysis of variance to test the effects of group (placebo vs. no-placebo) and time (before vs. after AoK).

All of the recruited 160 participants completed the study (no dropout). The students were not compensated for participating (see Supplementary Figure S1. CONSORT flow chart).

### 2.2. Procedure

The present study was approved by the ethics committee of the university and carried out following the Declaration of Helsinki. Each participant provided written informed consent. This study was preregistered on the open science framework (OSF https://osf.io/kh4db; 20 October 2021).

All participants first received an information sheet that briefly explained the program. It was stated that prosocial behaviors and happiness/satisfaction are positively associated with each other; the concept of an AoK was defined as a positive deed or gesture. Some examples were provided (e.g., giving a compliment, cooking for someone, or helping someone with a project). It was noted that AoKs are not about spending money.

Participants were randomly assigned (with a random number table) to the placebo group (*n* = 71) or the no-placebo group (*n* = 89). Both groups were asked to perform one act of kindness (AoK) per day for one week (seven AoKs in total). Participants were instructed to plan the AoKs (thus, the AoKs were entirely participant-defined), select a recipient, and rate their emotional state before and after performing the AoK (‘How do you feel right now?’; ‘How excited are you?’; ‘How satisfied are you?’; Likert scale ratings: 0 = not pleasant, calm, not satisfied; 100 = very pleasant, excited, satisfied). The AoK givers were free to choose the time of the day for the AoK, and the recipients were not previously informed that they had been chosen as targets. The participants used a smartphone app to write down the planned AoKs and for the evaluation of the AoKs after completion. The participants had no contact with each other during the intervention and were not informed about the different experimental conditions.

Participants of the placebo group received 30 mL of sunflower oil provided in a blue glass bottle with a dropper for oral administration. The label of the bottle stated ‘placebo’. Participants were instructed to take 3 drops of the oil per day in the morning. It was stated that the placebo would help them to complete the AoK. Moreover, the participants received written information that placebos made of an inert substance (like sugar pills) have been shown in scientific studies to produce significant improvement in various conditions (e.g., reduction in negative emotions, improvement of relaxation responses). It was further stated that deception is not necessary for the placebo effect to occur. Again, scientific studies were briefly described to underline this statement. Finally, a mechanism of placebo effects was mentioned (conditioning), which enables automatic responses to a placebo (an inert pill that looks like a pain killer that has been used before and reliably produced analgesic effects).

At the end of the one-week program, the participants were asked to rate the perceived effectiveness of the OLP (0 = not effective; 100 = very effective), and they returned the bottle (to check placebo intake).

Before and after the program, the participants completed the following questionnaires by means of an online survey tool:

1. The Positive Affect Negative Affect Schedule (PANAS; German version by Breyer and Bluemke [21]) is a self-report questionnaire that consists of 20 items to measure positive affect (e.g., I feel strong, active) and negative affect (e.g., I feel afraid, nervous). Each item is rated on a 5-point scale (1 = not at all; 5 = very much). McDonald’s omega in the present sample was 0.88 (positive affect) and 0.87 (negative affect).
2. The Satisfaction with Life Scale (German version [22]; McDonald’s omega = 0.85) has five items (e.g., ‘The conditions of my life are excellent.’ and ‘If I could live my life over, I would change almost nothing.’), which are answered on a 7-point Likert scale (1 = strongly disagree; 7 = strongly agree).
3. The Flourishing Scale (German version [23]; McDonald’s omega = 0.88) is an 8-item measure of self-perceived success in areas such as relationships, self-esteem, purpose, and optimism. The items (e.g., ‘I lead a purposeful and meaningful life.’, ‘amy social relationships are supportive and rewarding.’) are rated on a 7-point scale (1 = I absolutely do not agree, 7 = I absolutely agree).

### 2.3. Data Acquisition

The data gathering (conducted over seven days) was achieved by combining a PWA (Progressive Web App) and a remote server for storage. The server was encrypted using an SSL connection. Participants first installed the PWA on their mobile phones and then answered the questions before and after performing the AoKs. The survey was conducted via a webpage created with HTML, CSS, and Javascript (using the Vue.js Framework). The anonymous data were sent to a remote server where a Python Flask script handled the data collection and created a CSV file for each participant. The participants were instructed once how to use the app before the intervention; no further instructions or reminders were sent.

### 2.4. Statistical Analysis

To test the effects of the group (placebo, no-placebo) on AoK quantity (number of completed AoKs), a *t*-test was computed. A mixed-model analysis of variance (ANOVA) was performed to test the effect of Group and Time (before/after the daily AoK) on reported feelings of pleasantness, arousal, and satisfaction. An additional ANOVA tested the effect of the program (before/after the one-week program) and group on the questionnaire scores (positive affect/negative affect, satisfaction with life, flourishing). We report partial eta squared as an effect size measure. Exploratory correlation analyses were conducted for the OLP group to test the association between the perceived effectiveness of the placebo and the affective responses of the AoK givers.

In the total sample, the number of conducted AoKs varied between 1 and 11 (implying that some of the participants completed more than the required number of seven AoKs). None of the participants was excluded from the analysis.

## 3. Results

### 3.1. Acts of Kindness: Quantity

**Figure 1:**
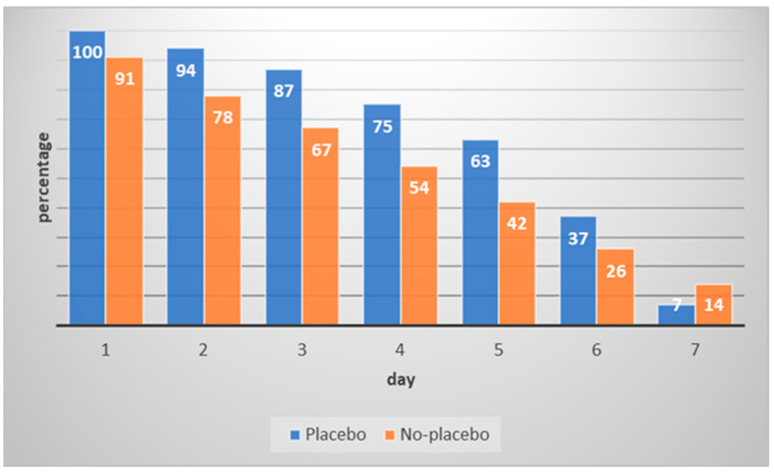
Percentage of completed acts of kindness per day and group.

The percentage of completed AoKs per day and group is depicted in Figure 1. Over the one-week interval, there was a strong decline in the number of AoKs. The placebo group completed more AoKs (M = 5.83, SD = 1.55) than the no-placebo group (M = 5.19, SD = 1.82, *t*(157.3) = 2.40, *p* = 0.018; Cohen’s d = 0.38; 95% CI [0.06, 0.69]). Eleven participants (7%) conducted more than the required seven AoKs (range: 8–11). Six participants were in the placebo group; five were in the no-placebo group.

The three most common acts of kindness included ‘providing food/drinks to others’ (e.g., giving a bar of chocolate to the other person; making dinner; buying someone a drink; 39%), ‘giving a compliment’ (13%), and ‘giving someone a call/visiting someone’; 10%). The AoK receivers were partners (32%), friends/fellow students (27%), and family members (19%).

### 3.2. Immediate Effects of AoKs on Feelings of Pleasantness, Arousal, and Satisfaction

Pleasantness: The ANOVA revealed a significant effect of Time (F(1,152) = 29.94, *p* < 0.001, ηp^2^ = 0.165). The effects of Group (F(1,152) = 0.99, *p* = 0.32, ηp^2^ = 0.006) and Group × Time were not significant (F(1,152) = 0.14, *p* = 0.71, ηp^2^ = 0.001). The participants felt more positive directly after engaging in an AoK than before (see Table 1; *t*(153) = −5.53, *p* < 0.001; Cohen’s d = −0.45; 95% CI [−0.61, −0.28]).

Arousal: The effects of Time (F(1,152) = 0.90, *p* = 0.77, ηp^2^ = 0.001), Group (F(1,152) = 3.09, *p* = 0.08, ηp^2^ = 0.02), and the interaction Group × Time (F(1,152) = 1.05, *p* = 0.31, ηp^2^ = 0.007) were not significant.

Satisfaction: The effects of Time (F(1,152) = 0.13, *p* = 0.91, ηp^2^ < 0.001), Group (F(1,152) = 2.15, *p* = 0.15, ηp^2^ = 0.014), and an the interaction Group × Time (F(1,152) = 1.30, *p* = 0.26, ηp^2^ = 0.009) were not significant.

### 3.3. Effects of Performing AoKs over One Week

Satisfaction With Life: The ANOVA revealed a significant effect for Time (F(1,156) = 8.52, *p* = 0.004, ηp^2^ = 0.052). The participants reported greater satisfaction with life after the AoK program (see Table 1; *t*(157) = −2.91, *p* = 0.004; Cohen’s d = −0.23; 95% CI [−0.39, −0.73]). The effects of Group (F(1,156) = 0.04, *p* = 0.84, ηp^2^ < 0.001) and Group × Time were statistically not significant (F(1,156) = 0.11, *p* = 0.74, ηp^2^ = 0.001).

Positive Affect/Negative Affect/Flourishing: None of the main effects and interaction effects were statistically significant (all *p* > 0.10; see Supplementary Materials, Table S1).

### 3.4. Correlation Analyses

The average rating for the perceived OLP effectiveness was M = 1.24 (SD = 1.64; observed range: 1–6). The effectiveness rating was not correlated with AoK-related changes in feelings of pleasantness (*r* = 0.13, *p* = 0.28) and satisfaction with life (*r* = 0.09, *p* = 0.44).

## 4. Discussion

The participants in the current study were asked to complete a daily act of kindness (AoK) over a period of one week, either with or without a non-deceptive placebo. In line with previous findings (e.g., [11,24]), the performance of AoKs was associated with increases in feelings of pleasantness and satisfaction with life. Directly after the AoK, participants felt more positive, and after one week of performing daily AoKs, their overall assessment of feelings and attitudes about life was also more positive.

However, over the course of the week, the motivation to carry out the AoKs decreased strongly. While almost all participants performed an AoK on the first day of the program, only 11 percent completed an AoK on the final day. In the majority of previous studies, the required quantity of AoKs was lower than in the current investigation (e.g., [11,25]). For instance, the ‘next door kindness challenge’ [25] involved doing (at least) one AoK per week over four weeks. Similarly, in a study by Chancellor et al. [11], participants carried out five AoKs within four weeks. In a study by Ouweneel et al. [19], however, a higher quantity of AoKs was used: university students were asked to perform at least five AoKs per day in an academic context (i.e., the AoKs had to take place at the university or had to be related to academic tasks). The authors mentioned no problems regarding adherence, but at the same time, compliance was not measured directly in that study (the participants provided written summaries). The app-assisted approach of the current study may have very likely resulted in more reliable data. Personal feedback from some of the participants included comments regarding experiencing pressure to carry out daily AoKs. Because they were instructed specifically to be nice to others, this reduced the perceived freedom of choice and resulted in reactance. Future studies on the effects of AoKs should also use an app-assisted approach to monitor compliance (problems).

In line with our hypothesis, the placebo group performed more AoKs (M = 5.8 per week) than the no-placebo group (M = 5.2 per week). Previous studies have demonstrated that placebos can increase the frequency of specific behaviors. For example, placebo treatment (with deceptive as well as non-deceptive instructions) has been associated with improved compliance in practicing relaxation training [6,10]. In studies on sports performance, participants who had received a placebo (a ‘performance enhancer’) displayed higher levels of endurance (for a review, see [8]). It has also been shown that placebos prescribed as ‘cognitive enhancers’ have improved the effects of cognitive training [26]. In summary, placebos have been shown to boost motivation in different contexts [9].

In the present investigation, the placebo did not cause an additional increment in subjective well-being induced using the AoK program in comparison to the no-placebo group. The placebo group and the no-placebo group did not differ concerning the AoK-related increase in reported feelings of pleasantness and satisfaction with life. This lack of difference might be related to the small increment in AoK quantity (+0.6 AoKs on average) induced using the placebo.

It has to be noted that the average effectiveness rating for the OLP was low. Thus, the placebo treatment was not perceived as being very helpful in supporting the engagement in AoKs. Traditional placebo research (with deceptive suggestions) has demonstrated that positive expectations are a central mechanism of the placebo response. However, non-deceptive placebo research has found that taking the placebo reliably is crucial, while positive expectations are helpful but not necessary [27,28]. At the same time, critical views regarding OLP treatment should also be taken into account. A study by Haas et al. [29] investigated outcome expectations and general acceptance of OLPs in the lay population. Compared to OLPs, the application of deceptive placebos (DP) was rated as more acceptable, and outcome expectations were higher for DPs compared to OLPs.

Investigations that directly compared deceptive vs. non-deceptive placebo treatment are still rare and have produced inconsistent findings. In a study on placebo analgesia, participants of a deceptive placebo group did not differ in their pain ratings from the OLP group, and both types of placebos were associated with pain reduction relative to a no-placebo group [30]. In another study [31], participants viewed a sad movie scene after placebo administration. Only the deceptive placebo but not the OLP reduced reported sadness. Two PMR investigations that analyzed the effects of a placebo (sunflower oil) used the same design and procedure with one exception: the oil was introduced as a natural medicine (deceptive placebo) in one study or as an OLP in the other study [6,10]. The placebos enhanced the practicing frequency compared to no-placebo. The deceptive placebo additionally improved the relaxation quality (feelings of reduced tension). This effect was not detected for the OLP. Therefore, a future investigation could test the effects of a wouldeceptive placebo’ to promote AoKs or could select participants with more positive attitudes toward non-deceptive placebo treatment.

It also has to be noted that the present study used a ‘take-home placebo’. The participants received a bottle of the placebo oil at the beginning of the study from the experimenter. The intake of the placebo had to be organized by the participants at home without receiving further instructions and feedback. The procedure was only guided using the smartphone application, which required giving emotional ratings directly before and after the AoK. However, it has been demonstrated that the social factors surrounding the administration of a placebo are important to obtain effects [28]. Typically, an OLP treatment takes place within the context of patient-clinician engagement. Kaptchuk [28] suspected that the absence of verbal and nonverbal cues (as provided by speech, appearance, and ritualized behaviors of the placebo provider) that lead to trust and rapport would reduce the effectiveness of an OLP. These cues are not present if a placebo is self-administered at home. However, this type of treatment is similar to other forms of treatment (e.g., with prescription medicine). Thus, this approach is ecologically valid. Often, OLP research has focused on participants’ responses to a single-dose placebo in the laboratory (e.g., [4,5]). This is an artificial environment that does not mirror situations in daily life. A previous study already demonstrated the positive effects of a deceptive take-home placebo on self-reported stress, anxiety, and symptoms of depression in a non-patient sample [32]. The participants received an ‘anti-stress treatment spray’ for self-administration over three days. The specific effects observed (i.e., reduction in stress vs. depression) were associated with the labeling of the placebo (as oxytocin vs. serotonin).

## 5. Limitations

We need to mention the following limitations of this study. First, we investigated a specific group of AoK givers (university students) and AoK receivers (friends/family). Therefore, the present findings cannot be generalized to other groups or types of prosocial actions (e.g., random acts of kindness involving strangers).

Second, we did not assess the responses of the AoK recipients. Their reactions (e.g., positive feedback vs. lack of positive feedback) may have influenced the affective response of the AoK givers.

Third, the duration of the AoK program may need to be extended (longer than one week), and the quantity of AoKs required reduced to possibly increase compliance.

Fourth, the sample was characterized by an uneven ratio of male and female participants (125 females vs. 35 males). This needs to be acknowledged as a limitation of the study, considering well-established sex differences in prosocial behaviors and psychosocial well-being [33].

Finally, this study looked at the immediate effects of kindness on well-being. Future studies should also include more distal measures to analyze the long-term effects of AoKs.

## 6. Conclusions

This study demonstrated the positive effects of an OLP on the number of acts of kindness performed. Thus, placebo treatment can promote the motivation to engage in AoKs.

## Figures and Tables

**Table 1:** Means (standard deviations) for affective ratings and questionnaire scores before and after doing acts of kindness (AoK), *t*-tests (*p*-values), 95% confidence intervals [CI]).

	Placebo Group M (SD)	No-Placebo Group M (SD)	*t* (*p*)	CI	Total Group M (SD)
**Feelings of Pleasantness**					
Before AoK	59.02 (13.29)	56.52 (15.40)	1.07 (0.29)	−2.12, 7.12	57.67 (14.47)
After AoK	63.27 (13.71)	61.39 (15.34)	0.79 (0.43)	−2.79, 6.54	62.26 (14.60)
**Arousal**					
Before AoK	24.82 (13.25)	27.81 (16.05)	−1.25 (0.11)	−7.73, 1.74	26.43 (14.85)
After AoK	23.43 (15.45)	28.57(17.31)	−1.93 (0.06)	−10.39, 0.14	26.20 (16.62)
**Satisfaction**					
Before AoK	52.83 (13.21)	52.25 (13.17)	0.27 (0.39)	−3.66, 4.82	52.52 (13.15)
After AoK	54.60 (11.45)	50.80 (12.62)	1.93 (0.06)	−0.83, 7.68	52.57 (12.20)
**Satisfaction with Life**					
Before program	5.13 (1.19)	5.11 (0.96)	0.09 (0.93)	−0.32, 0.35	5.12 (1.07)
After program	5.30 (1.10)	5.25 (0.98)	0.45 (0.65)	−0.25, 0.40	5.27 (1.03)
**Positive Affect**					
Before program	27.78 (7.46)	28.38 (7.07)	−0.52 (0.61)	−2.91, 1.71	28.12 (7.23)
After program	28.71 (7.35)	27.94 (7.54)	0.81 (0.42)	−1.39, 3.24	28.28 (7.44)
**Negative Affect**					
Before program	17.20 (6.25)	17.43 (6.82)	−0.22 (0.83)	−2.32, 1.86	17.33 (6.55)
After program	16.48 (6.56)	16.70 (6.07)	−0.26 (0.79)	−2.23, 1.71	16.61 (6.27)
**Flourishing**					
Before program	5.67 (0.99)	5.64 (0.79)	0.33 (0.74)	−0.23, 0.33	5.66 (0.88)
After program	5.73 (0.90)	5.60 (0.82)	1.10 (0.27)	−0.12, 0.42	5.66 (0.86)

## Data Availability

On request by the corresponding author.

## Supplementary Materials

The following supporting information can be downloaded at: https://www.mdpi.com/article/10.3390/bs13090703/s1. Figure S1. CONSORT flow chart, Table S1. Analyses of variance—nonsignificant effects.
