# Supplementary material for: Non-Deceptive Placebos Can Promote Acts of Kindness: A Randomized Controlled Trial
Source: Behav Sci (Basel). 2023 Aug 23;13(9):703. doi: 10.3390/bs13090703 (PMC10525077; doi:10.3390/bs13090703)
Supplement: Supplementary file 1 [file behavsci-13-00703-s001.zip › behavsci-2492361-supplementary.pdf]

behavioral sciences

MDPI

Supplementary Material

Non-deceptive Placebos Can Promote Acts of Kindness: A Randomized Controlled Trial

Anne Schienle * and Isabella Unger

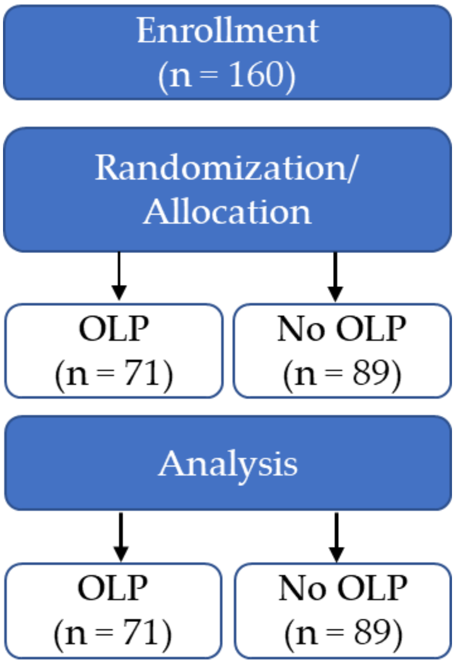

Figure S1. CONSORT flow chart. Footnote: OLP: open-label placebo.

Table S1. Analyses of variance - nonsignificant effects.

**Positive Affect**
Time ($F(1,156) = 0.20$, $p = 0.652$, $\eta p^2 = 0.001$)
Group x Time ($F(1,156) = 1.63$, $p = 0.203$, $\eta p^2 = 0.010$)
Group ($F(1,156) = 0.006$, $p = 0.938$, $\eta p^2 < 0.001$)
**Negative Affect**
Time ($F(1,156) = 2.58$, $p = 0.110$, $\eta p^2 = 0.016$)
Group x Time ($F(1,156) = 0.001$, $p = 0.998$, $\eta p^2 < 0.001$)
Group ($F(1,156) = 0.006$, $p = 0.807$, $\eta p^2 < 0.001$)
**Flourishing**
Time ($F(1,156) = 0.35$, $p = 0.851$, $\eta p^2 < 0.001$)
Group x Time ($F(1,156) = 1.63$, $p = 0.204$, $\eta p^2 = 0.010$)
Group ($F(1,156) = 0.38$, $p = 0.537$, $\eta p^2 < 0.002$)
